# Method for Vibration Response Simulation and Sensor Placement Optimization of a Machine Tool Spindle System with a Bearing Defect

**DOI:** 10.3390/s120708732

**Published:** 2012-06-27

**Authors:** Hongrui Cao, Linkai Niu, Zhengjia He

**Affiliations:** State Key Laboratory for Manufacturing Systems Engineering, Xi'an Jiaotong University, Xianning West Road, Xi'an, 710049, China; E-Mails: linkai-n@163.com (L.N.); hzj@mail.xjtu.edu.cn (Z.H.)

**Keywords:** bearing defects, vibration simulation, finite element model, sensor placement optimization, spindle

## Abstract

Bearing defects are one of the most important mechanical sources for vibration and noise generation in machine tool spindles. In this study, an integrated finite element (FE) model is proposed to predict the vibration responses of a spindle bearing system with localized bearing defects and then the sensor placement for better detection of bearing faults is optimized. A nonlinear bearing model is developed based on Jones' bearing theory, while the drawbar, shaft and housing are modeled as Timoshenko's beam. The bearing model is then integrated into the FE model of drawbar/shaft/housing by assembling equations of motion. The Newmark time integration method is used to solve the vibration responses numerically. The FE model of the spindle-bearing system was verified by conducting dynamic tests. Then, the localized bearing defects were modeled and vibration responses generated by the outer ring defect were simulated as an illustration. The optimization scheme of the sensor placement was carried out on the test spindle. The results proved that, the optimal sensor placement depends on the vibration modes under different boundary conditions and the transfer path between the excitation and the response.

## 1. Introduction

Due to the increasing demands from aerospace, automotive, die/mold and other industries, machine tools with higher speed, precision and reliability are required urgently. In a machine tool, the spindle directly affects the cutting ability of the whole machine tool, since it either carries cutting tools as in milling operations, or work-pieces as in turning. In general, there are four types of spindle depending on the type of drives used: belt drive, gear drive, direct drive and integrated (built-in) drive [1]. The belt-driven spindles and gear-driven spindles are widely used in conventional machining, while the direct drive spindles and integrated spindles can run at higher rotation speeds, from 15,000 rpm. Recently, Abele *et al.* [2] have provided a detailed review on the historical development, recent challenges and future trends of machine tool spindles. Mechanical design of spindles, modeling the thermal and dynamical behavior of spindle units, sensor and actuator integration, were addressed in detail. In the application, the condition monitoring and fault diagnosis are very important to maintain the performance of spindles. Unexpected failure of spindles can lead to severe part damage and costly machine downtime, affecting the overall production logistics and productivity [3].

Bearings play an important role in machine tool spindle systems. Compared with hydrostatic, aerostatic or magnetic bearings [4], rolling element bearings are still most commonly used today in the spindles, which can provide the required precision, load carrying capacity, and spindle speeds. The bearings are usually extremely reliable, but failure can still occur by improper operation, overloading and thermal seizure. The early stage of bearing damage is often characterized by a sizeable local defect (pits or spalls) on inner raceway, outer raceway or rolling balls. When this occurs, subsequent rolling over of the damage will produce repetitive shocks or short-duration impulses. Extensive work has been carried out on the detection and diagnosis of bearing faults over the past several decades, as reviewed in several papers [5–7]. There is considerable interest in diagnostics and prognostics of rolling element bearings based on vibration analysis and signal processing [8]. Many powerful diagnostic methods have been developed to detect bearing failures based on advanced signal processing techniques such as envelope analysis [9,10], wavelets [11–13], empirical mode decomposition (EMD) [14–16], spectral kurtosis [17–20], and information entropy [21,22].

Most studies have focused on feature extraction of the faulty bearing from vibration signals, however, just a few of works attempted to model bearing faults mathematically so that the vibration signals generated by the defects can be simulated and explained theoretically. McFadden and Smith [23] gave a pioneering model to simulate the vibration signals produced by localized defects of rolling ball bearings. The successive impacts generated by a defect during rotation were modeled as a periodic impulse series. Based on this work, Wang and Kootsookos [24] proposed a general model for faulty bearing signals, which contained effects caused by non-uniform load distribution, machinery induced vibration and measurement noise. In another aspect, Ho and Randall [25] improved McFadden and Smith's model by incorporating slight random variations in the spaces of the excitation pulses. They pointed out that the random fluctuation causes the harmonic frequency components to smear laterally and this viewpoint gave a good explanation of the vibration spectra observed in reality. The bearing fault signals were generated as a series of impulse responses of a single degree of freedom (DOF) system. Similarly, Brie [26] also concerned the non-strict periodicity of the impacts induced by the defect and a concept called quasi-periodic impulse train was proposed for the impact signals. The bearing is modeled as a mass-spring-damper system with time-varying parameters. Later, Antoni and Randall [27] found out that vibration signals generated by localized faults were not exactly quasi-periodic since the random slippages are non-stationary and consequently developed a stochastic model to describe the vibration signals generated by localized defects. They treated the repetitive impacts as a non-stationary process for the first time. The vibration signals were viewed as the response of a linear system which is time-varying and accommodate some degrees of stochasticity. Choudhury and Tandon [28] modeled the rotor-bearing system as a 3-DOF system, and the vibration response due to a localized defect under radial load conditions were predicted with the model.

In the above research, rolling element bearings are modeled linearly and usually simplified as single DOF systems. However, due to the nonlinear Hertzian force/deformation relationship, the varying compliance vibration effect, and lubricant film effect, rolling ball bearings show high non-linearity and time-varying characteristics during operation, so a linear model is not adequate to express the dynamic behavior of bearings. Recently, Sopanen and Mikkola [29,30] developed a dynamic nonlinear model of deep groove ball bearings, which included the effects of non-linear Hertzian contact deformation and elastohydrodynamic fluid film. Distributed defects and localized defects were both taken into consideration. Sawalhi and Randall [31,32] presented an integrated simulation model for a gearbox test rig through the incorporation of a time-varying, non-linear stiffness bearing model into a developed gear model. The incorporated bearing model has the capacity to simulate both localized spalls (inner race, outer race and rolling balls) and extended inner and outer race faults (rough surfaces). Similarly, Rafsanjani *et al.* [33] proposed an analytical model to study the nonlinear dynamic behavior of rolling ball bearing systems including surface defects. Mathematical expressions were derived for local defects of inner race, outer race and rolling balls. Sawalhi and Randall [8] simulated the acceleration time signal responses resulting from a rolling element entry into and exit from a typical spall, which has the potential to enable quantification of the fault size.

Bearings in rotating machines are mechanically coupled to supporting structures, and thus defect-induced transient signals are often masked by interfering signals or background noise [34]. When bearing defects occur, the generated impacts can cause system vibration at many frequencies from different structures. The measured vibration responses to the localized defects are affected by the transmitting media between defects and sensors. However, most existing fault models don't contain the effects induced by other components other than bearings.

In the machine tool spindle system, bearings work as a subsystem. Accurately predicting the vibration signals of the spindle with bearing defects remains a challenging task because of its complicated nonlinear behavior. In this paper, the machine tool spindle system is focused on and an integrated finite element (FE) model for a spindle-bearing system is proposed. The nonlinear bearing model is developed based on Jones' bearing theory [35], while the drawbar, shaft and housing are modeled as Timoshenko's beam. The bearing model is integrated into the FE model of drawbar/shaft/housing by assembling equations of motion. In this way, bearings are coupled with other spindle components. The dynamic model is validated on a test spindle and then used to simulate the vibration signals generated by localized defects of the bearing outer ring. Considering the influences of the transmitting media between defects and sensors, the dynamic responses at different positions are compared to obtain an optimal sensor placement strategy.

The rest of the paper is organized as follows: in Section 2, a dynamic model of a spindle-bearing system is given with experimental validation. Localized bearing faults modeling is presented in Section 3, followed by the optimization of sensor positions in Section 4. The conclusions are given in Section 5.

## 2. Dynamic Model of a Spindle-Bearing System

**Figure 1.**
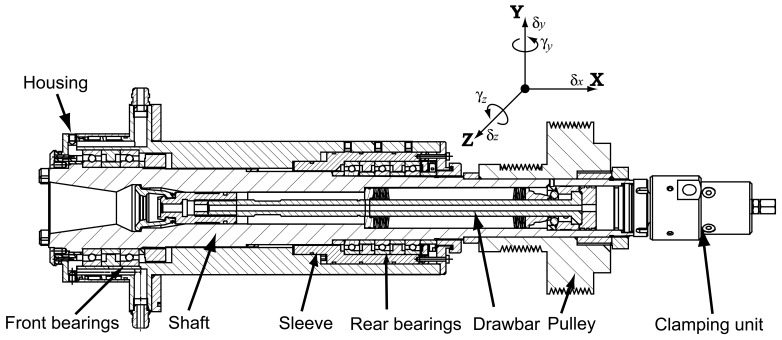
The test spindle.

The machine tool spindle system usually consists of drawbar, shaft, housing, bearings and other accessories. A typical test spindle is shown in Figure 1, which has five angular contact ball bearings in an O-type configuration. This spindle was used in [36–38] to demonstrate the FE modeling process as well.

The spindle drawbar/shaft/housing and the angular contact ball bearing are modeled separately. The drawbar, shaft, and housing are modeled as Timoshenko's beam, while the nonlinear bearing model is developed based on Jones' bearing theory which considers the centrifugal force and gyroscopic effects from rolling balls. The bearing model is integrated into the FE model of shaft/housing by assembling equations of motion. The pulley, clamping unit and other accessories are modeled using rigid disk elements. The spacers between bearings are modeled using bar elements. This FE modeling process can be extended to high-speed spindles of integrated structure, in which the motor is modeled using rigid disk elements. With the FE model of the spindle, the Newmark integration method is used to obtain the vibration responses numerically.

### 2.1. Drawbar/Shaft/Housing Modeling

**Figure 2.**
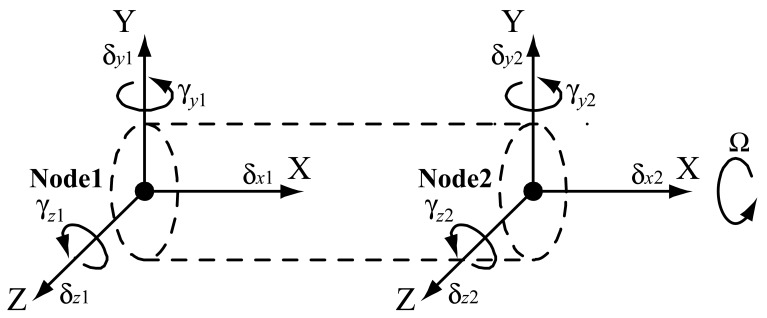
Timoshenko beam element.

The Timoshenko beam element (as shown in Figure 2) is used to model the drawbar, shaft and housing. The motion of each node ***q*** is described by three translational (*δ_x_, δ_y_, δ_z_*) and two rotational (*γ_y_, γ_z_*) degrees of freedom:

(1) $$\text{q}={\left\{\delta_{x},\delta_{y},\delta_{z},\gamma_{y},\gamma_{z}\right\}}^{T}$$

where the torsional motion is not included.

The equation of motion for the drawbar/shaft in matrix form is expressed by including the centrifugal force and gyroscopic effects as:

(2) $${\text{M}}_{b}\ddot{\text{x}}-\Omega {\text{G}}_{b}\dot{\text{x}}+({\text{K}}_{b}-\Omega^{2}{\text{M}}_{C})\text{x}={\text{F}}_{b}$$

where ***K****_b_* and ***M****_b_* are the stiffness and mass matrices of beam elements, ***G****_b_* is the skew-symmetric gyroscopic matrix of the rotating shaft, ***M****_C_* is the mass matrix for the centrifugal force effect on the shaft, Ω is the rotating speed, and ***F****_b_* is the external force vector. The subscript *b* represents the beam. For the housing, Equation (2) is still applicable. The rotating speed Ω is set to zero as the housing is stationary.

In the spindle system, disk and other accessories (e.g., clamping units, nuts) are commonly used, and they are modeled using rigid disk elements. The equation of motion is given as:

(3) $${\text{M}}_{d}\;\ddot{\text{x}}-\Omega {\text{G}}_{d}\;\dot{\text{x}}={\text{F}}_{d}$$

where ***M****_d_* is the mass matrix of disk elements, ***G****_d_* is the gyroscopic matrix and ***F****_d_* is the external force vector.

### 2.2. Nonlinear Bearing Model

**Figure 3.**
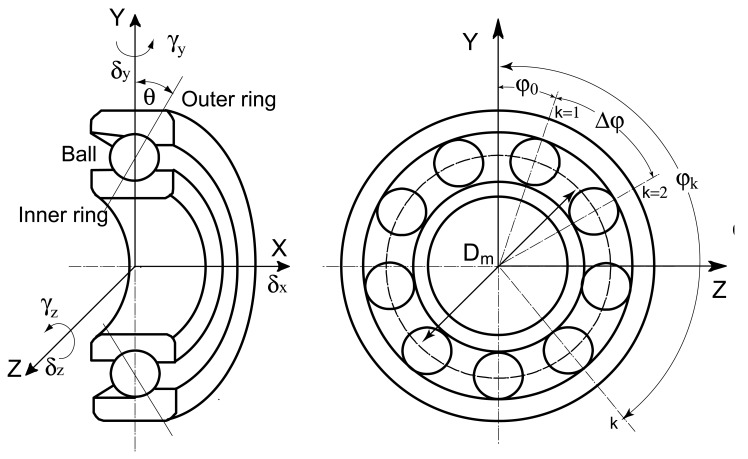
Geometry of an angular contact ball bearing.

The geometric drawing of an angular contact ball bearing is shown in Figure 3. The radii of the inner and outer ring grooves are:

(4) $$\begin{array}{cc} r_{i}=f_{i}\cdot D, & \;r_{o}=f_{o}\cdot D, \end{array}$$

where *f_i_* and *f_o_* are curvature ratios of the inner ring and the outer ring, *D* is the diameter of the bearing ball. The angular position of the *k*_th_ ball is:

(5) $$\begin{array}{cc} \varphi_{k}=\varphi_{0}+2\pi (k-1)/N, & \;k=1,2,\ldots ,N. \end{array}$$

Similar to the shaft/housing, the bearing is modeled with ‘bearing element’. Each ‘bearing element’ consists of two nodes—the inner ring node and the outer ring node. The motion vectors of bearing nodes are expressed by ***q****_i_* (the inner ring node) and ***q****_o_* (the inner ring node), respectively:

(6) $$\begin{array}{cc} {\text{q}}_{i=}{\left\{\delta_{xi},\delta_{yi},\delta_{zi},\gamma_{yi},\gamma_{zi}\right\}}^{T}, & {\text{q}}_{o=}{\left\{\delta_{xo},\delta_{yo},\delta_{zo},\gamma_{yo},\gamma_{zo}\right\}}^{T}, \end{array}$$

**Figure 4.**
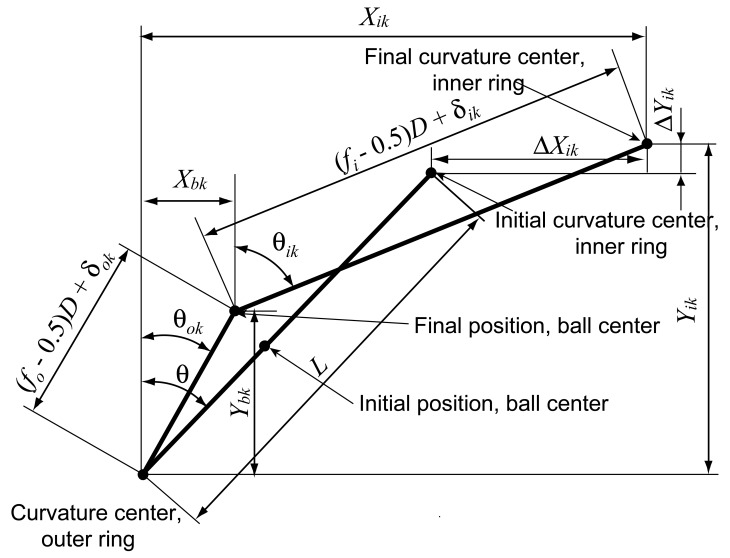
Positions of ball center and raceway groove curvature centers.

Under the working condition, the bearing rotates at high speed with applied axial loads and/or radial loads and the relative positions between the inner ring, ball and outer ring change consequently. Based on Jones' bearing model, the geometry relations of the bearings in the X-Y plane are shown in Figure 4.

From Figure 4, the following geometric equations can be obtained using the Pythagorean Theorem:

(7) $$\{\begin{array}{l} {\left(X_{ik}-X_{bk}\right)}^{2}+{\left(Y_{ik}-Y_{bk}\right)}^{2}-{\left[\left(f_{i}-0.5\right)+D+\delta_{ik}\right]}^{2}=0\\ {X_{bk}}^{2}+{Y_{bk}}^{2}-{\left[\left(f_{o}-0.5\right)D+\delta_{ok}\right]}^{2}=0 \end{array}$$

where *X_ik_* = *L*sin*θ* + Δ*X_ik_*, Δ*Y_ik_* = *L*cos*θ* + Δ*Y_ik_, δ_ik_* is the ball-inner raceway contact deformation, and *δ_ok_* is the ball-outer raceway contact deformation.

**Figure 5.**
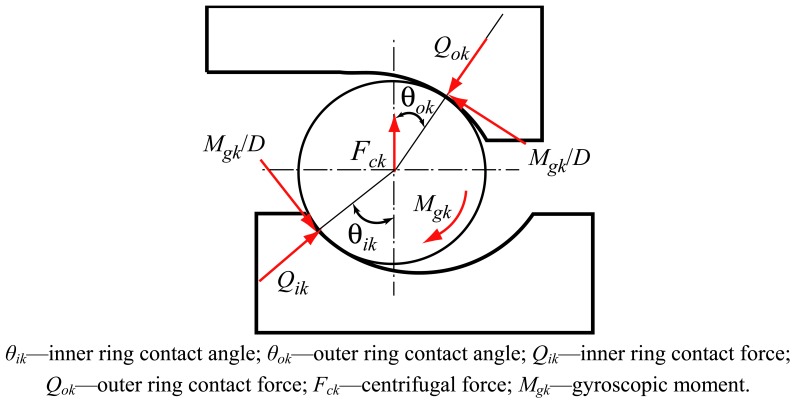
The force acting on the ball at angular position *φ_k_*.

Considering the plane passing through the bearing axis and the center of a ball located at azimuth *φ_k_* (see Figure 3), the load diagram without consideration of noncoplanar friction forces is obtained, as shown in Figure 5.

From Figure 5, the equilibrium of forces in the horizontal and vertical direction are given as:

(8) $$\{\begin{array}{l} Q_{ok}\cos\theta_{ok}-\frac{M_{gk}}{D}\sin\theta_{ok}-Q_{ik}\cos\theta_{ik}+\frac{M_{gk}}{D}\sin\theta_{ik}-F_{ck}=0\\ Q_{ok}\sin\theta_{ok}+\frac{M_{gk}}{D}\cos\theta_{ok}-Q_{ik}\sin\theta_{ik}-\frac{M_{gk}}{D}\cos\theta_{ik}=0 \end{array},$$

Combining Equation (7) and Equation (8), the following equations are obtained:

(9) $$\{\begin{array}{l} {\left(X_{ik}-X_{bk}\right)}^{2}+{\left(Y_{ik}-Y_{bk}\right)}^{2}-{\left[\left(f_{i}-0.5\right)D+\delta_{ik}\right]}^{2}=\varepsilon_{1}\\ {X_{bk}}^{2}+{Y_{bk}}^{2}-{\left[\left(f_{o}-0.5\right)D+\delta_{ok}\right]}^{2}=\varepsilon_{2}\\ Q_{ok}\cos\theta_{ok}-\frac{M_{gk}}{D}\sin\theta_{ok}-Q_{ik}\cos\theta_{ik}+\frac{M_{gk}}{D}\sin\theta_{ik}-F_{ck}=\varepsilon_{3}\\ Q_{ok}\sin\theta_{ok}+\frac{M_{gk}}{D}\cos\theta_{ok}-Q_{ik}\sin\theta_{ik}-\frac{M_{gk}}{D}\cos\theta_{ik}=\varepsilon_{4} \end{array}$$

where *ε*_1_, *ε*_2_, *ε*_3_ and *ε*_4_ are error variables. The Newton-Raphson iteration method is used to solve the Equation (9) and the values of *X_bk_, Y_bk_, δ_ik_, δ_ok_* can be found. Then the contact loads are calculated by Hertzian theory for spherical contact as:

(10) $$\begin{array}{cc} Q_{ik}=K_{i}\delta_{ik}^{3/2}, & Q_{ok}=K_{o}\delta_{ok}^{3/2} \end{array}$$

where *K_i_* and *K_o_* are the load-deflection coefficients.

The forces. ***F**_i_* = {*F_xi_, F_xi_, F_zi_, M_yi_, M_zi_*,}^T^ applied on the inner ring of the bearing are given as:

(11) $$\{\begin{array}{l} F_{xi}=\sum_{k=1}^{N}\left(Q_{ik}\sin\theta_{ik}+\frac{M_{gk}}{D}\cos\theta_{ik}\right)\\ F_{yi}=\sum_{k=1}^{N}\left(Q_{ik}\cos\theta_{ik}-\frac{M_{gk}}{D}\sin\theta_{ik}\right)\cos\varphi_{k}\\ F_{zi}=\sum_{k=1}^{N}\left(Q_{ik}\cos\theta_{ik}-\frac{M_{gk}}{D}\sin\theta_{ik}\right)\sin\varphi_{k}\\ M_{yi}=+\sum_{k=1}^{N}\left\{r_{ic}\left(Q_{ik}\sin\theta_{ik}+\frac{M_{gk}}{D}\cos\theta_{ik}\right)-f_{i}M_{gk}\right\}\sin\varphi_{k}\\ M_{zi}=-\sum_{k=1}^{N}\left\{r_{ic}\left(Q_{ik}\sin\theta_{ik}+\frac{M_{gk}}{D}\cos\theta_{ik}\right)-f_{i}M_{gk}\right\}\cos\varphi_{k} \end{array}.$$

Similarly, the forces ***F****_o_* = {*F_xo_, F_xo_, F_zo_, M_yo_, M_zo_*}^T^ applied on the outer ring of the bearing can be obtained. The bearing stiffness matrix is obtained by calculating the derivative of the force ***F*** = {*F_x_, F_x_, F_z_, M_y_, M_z_*}^T^ acting on the bearing rings with respect to the displacement ***q*** = {*δ_x_, δ_y_, δ_z_, γ_y_, γ_z_*}^T^ of bearing rings, namely:

(12) $${\text{K}}_{\text{B}}=\frac{\partial {\text{F}}_{i}}{\partial {\text{q}}_{i}}=\frac{\partial {\text{F}}_{o}}{\partial {\text{q}}_{o}}$$

The bearing stiffness matrix ***K***_B_ has the form:

(13) $${\text{K}}_{\text{B}}=\left[\begin{array}{lllll} k_{xx} & k_{xy} & k_{xz} & k_{x\theta_{x}} & k_{x\theta_{y}}\\ k_{yx} & k_{yy} & k_{yz} & k_{y\theta_{x}} & k_{y\theta_{y}}\\ k_{zx} & k_{zy} & k_{zz} & k_{z\theta_{x}} & k_{z\theta_{y}}\\ k_{\theta_{x}x} & k_{\theta_{x}y} & k_{\theta_{x}z} & k_{\theta_{x}\theta_{x}} & k_{\theta_{x}\theta_{y}}\\ k_{\theta_{y}x} & k_{\theta_{y}y} & k_{\theta_{y}z} & k_{\theta_{y}\theta_{x}} & k_{\theta_{y}\theta_{y}} \end{array}\right]$$

where *k_xx_* is the axial stiffness, *k_yy_* and *k_zz_* are the radial stiffness, *k_θxθx_* and *k_θyθy_* are the angular stiffness, and other terms are coupled stiffness.

### 2.3. Finite Element Model of the Spindle-Bearing System

**Figure 6.**
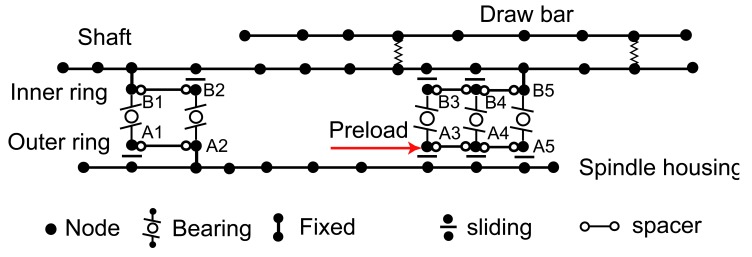
The finite element model of the spindle-bearing system.

The finite element model of the spindle-bearing system is shown in Figure 6. The black dots represent nodes, where each node has three translational and two rotational degrees of freedom. The connections between the drawbar and shaft are modeled by spring elements. The displacement relations among the spindle shaft, bearings, and the housing correspond to the configuration and preload mechanism of bearings [39]. For the test spindle (as seen in Figure 1), the five bearings are preloaded by a hydraulic system. Therefore, The inner ring of the first bearing (B1), the outer ring of the second bearing (A2), and the inner ring of the fifth bearing (B5) are fixed to the corresponding nodes of the shaft or housing. Other rings of the five bearings are sliding in axial direction.

By assembling the equations of the drawbar/shaft/housing and bearings, the following general nonlinear dynamic equation for the spindle-bearing system is obtained:

(14) $$\text{M}\ddot{\text{x}}+\text{C}\dot{\text{x}}+\text{Kx}=\text{F}$$

where ***M*** = ***M***_b_, ***C*** = ***C****_s_* − Ω***G****_b_*, ***K*** = ***K****_b_* + ***K***_B_ − Ω^2^***M****_C_* are the mass, damping and stiffness matrices. ***C****_s_* is the structural damping matrix constructed from modal damping ratios identified experimentally. The bearing stiffness matrix ***K***_B_ depends on the displacement, ***x*** among other factors. In turn, the displacement is affected by the system stiffness and the external force ***F***. Therefore, the dependency of bearing stiffness matrix on displacement is the root cause of the nonlinearity in the spindle system. The details of the matrices (***M***, ***C***, ***K***) can be referred in [40].

If the external load is time-varying, then the system responses are time-varying as well and the equation of motion at time *t* + Δ*t* is given as:

(15) $$\text{M}{\ddot{\text{x}}}_{t+\Delta t}+\text{C}{\dot{\text{x}}}_{t+\Delta t}+\text{K}{\text{x}}_{t+\Delta t}={\text{F}}_{t+\Delta t}.$$

Assuming that the displacement, velocity, and acceleration vectors at time *t* (*t* ≥ 0) are known, the time history responses of the spindle system (*i.e.*, displacement ***x***_*t*+Δ*t*_, velocity ***ẋ***_*t*+Δ*t*_ and acceleration ***ẍ***_*t*+Δ*t*_) at time *t* + Δ*t* can be evaluated numerically using the Newmark time integration method.

### 2.4. Model Validation

**Figure 7.**
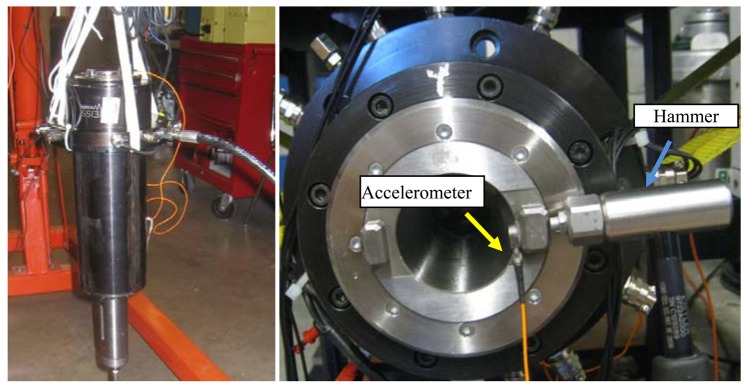
The hammer test of the spindle. (**a**) The test spindle under free-free condition. (**b**) The measured position.

The test spindle was hung using elastic strings as a free-free system. The impact forces were applied by a hammer on the spindle nose in the radial direction and the vibration responses at the opposite part of the nose were recorded by an accelerometer, as shown in Figure 7. The frequency response function (FRF) were measured by CutPro-MalTF® Fourier Analyzer.

In the simulation, some parameters must be identified. These parameters include joint stiffness between the drawbar and the shaft, and the system damping ratios. It is possible to obtain a good match between the simulation and the measurement by tuning these parameters. The joint stiffness was found to be 2 × 10^8^ N/m in the radial direction. The modal damping ratios were borrowed from experimental data and used in the FE simulations. From the simulated FRFs, the first mode ( $\omega_{n1}^{s}=965\;\text{Hz}$) and fourth mode ( $\omega_{n4}^{s}=2,722\;\text{Hz}$) have been found to be the most dominant. Corresponding to these two main modes, experimental model damping ratios of 1.6% ( $\omega_{n1}^{x}=955\;\text{Hz}$) and 2.9% ( $\omega_{n4}^{s}=2,745\;\text{Hz}$) were used in the simulation. Damping ratios of other modes were set as 3%.

**Figure 8.**
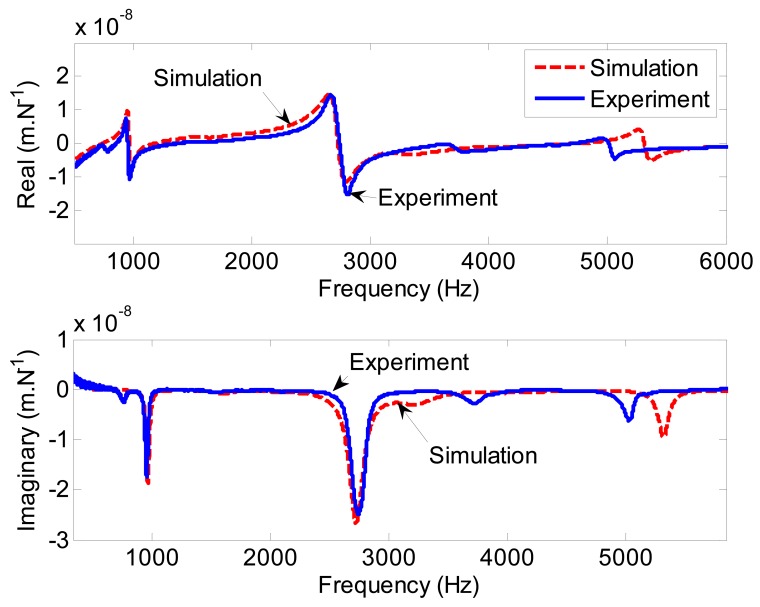
Simulated and experimentally measured FRFs at the spindle nose in the free-free condition.

The simulated and experimentally measured FRFs at the spindle nose are shown in Figure 8. On the whole, the simulated and experimental FRFs are in reasonable agreement, which indicates the validity of the spindle-bearing model.

**Figure 9.**
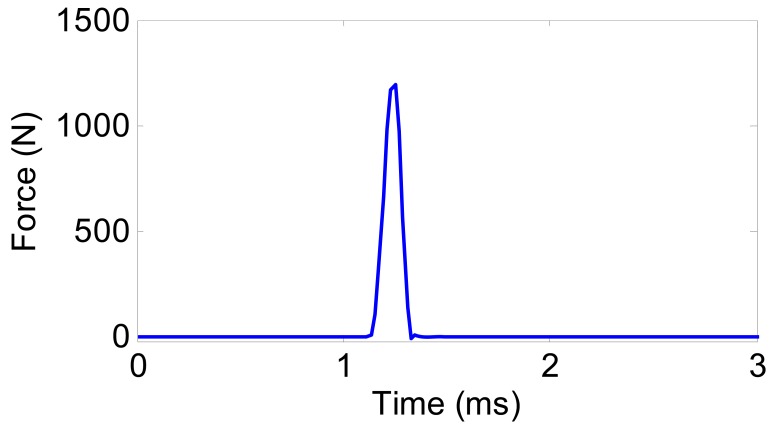
The measured impact force at the spindle nose.

The time-history response of the acceleration ***ẍ*** due to the impact force is investigated as well. In the simulation, the measured impact force shown in Figure 9 was input to the spindle-bearing model. This is a typical impulse force whose duration is about 0.24 ms.

**Figure 10.**
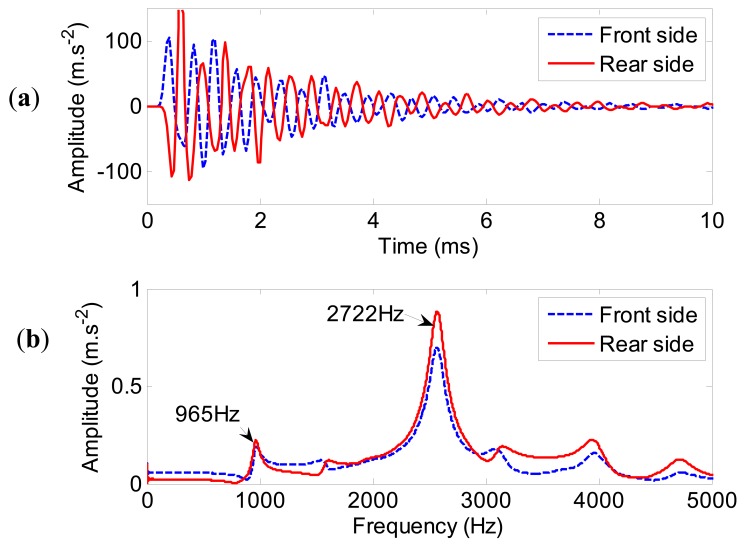
The acceleration time responses and spectra at the front and the rear side of the spindle housing: (**a**) the acceleration time responses, (**b**) the spectra.

The acceleration time responses and their spectra at the front side and the rear side of the spindle housing are predicted in Figure 10(a,b). The phases of the two acceleration time responses are converse (Figure 10(a)). From Figure 10(b), it can be seen that there are two distinct peaks in the frequency range of 0–5,000 Hz, which are corresponding to the two dominant modes of the spindle, *i.e.*, the first mode ( $\omega_{n1}^{s}=965\;\text{Hz}$) and the fourth mode ( $\omega_{n4}^{s}=2,722\;\text{Hz}$). Therefore, the predicted acceleration time responses at both the front side and the rear side of the spindle housing are acceptable. The FE model of the test spindle is capable of predicting the acceleration time responses due to the excitation.

If bearing defects occur in the spindle system, the defect in one surface of a bearing strikes another surface and then an impact force is generated, which may excite resonances in the spindle-bearing system. With the input excitation from an appropriate bearing fault model, the simulation of vibration response caused by the bearing defects is feasible.

## 3. Localized Bearing Defects Modeling and Vibration Simulation

Vibration signals measured on a spindle contain rich physical information about the operating conditions. When local defects (e.g., cracks, pits, spalls) exist in one of the bearing components, transient impact forces occur whenever such a defect on one surface strikes its mating surface. The impact forces will excite the vibration responses at the natural frequencies of bearing parts and housing structure. During the rotating process, a series of approximately equally spaced force impulses are produced. The repetition rate of the force impulses is equal to the characteristic frequencies of the bearing, *i.e.*, ball passing frequency for the outer raceway, ball passing frequency for the inner raceway, and twice the ball spin frequency [41].

**Figure 11.**
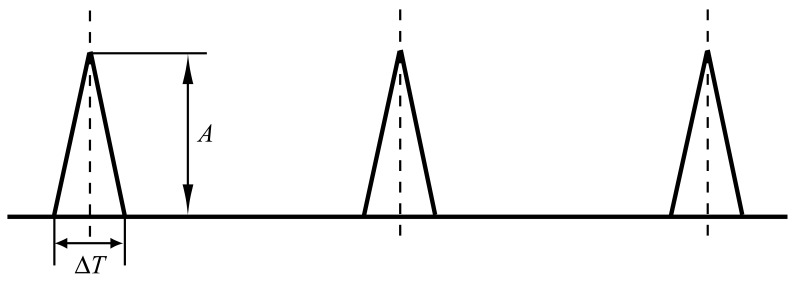
The force impulse form.

Similar to the impact force generated by a hammer test, the shape of the force impulse is modeled as a triangular form, as shown in Figure 11. In practice, the pulse form may not be of such a regular shape. The duration time Δ*T* can be determined by dividing the defect width (*L*) by the relative velocity (*v_r_*) between the mating elements, *i.e.*, Δ*T* = *L*/*v_r_*. The magnitude *A* of the impulse is affected by both the contact load and the angular velocity at the defective surface, which can also be modulated due to relative motion of the load zone. The accurate calculation of the magnitude *A* is beyond the scope of this paper, which will be addressed in the future work.

To explain the localized bearing faults modeling process, the outer ring defect of the first bearing (HYKH61914) in the spindle is modeled as an example. The bearing parameters are listed in Table 1. With the expressions given in [41], the outer race defect frequency is calculated as 298 Hz when the rotating speed of the spindle is 1,200 rev/min.

**Figure 12.**
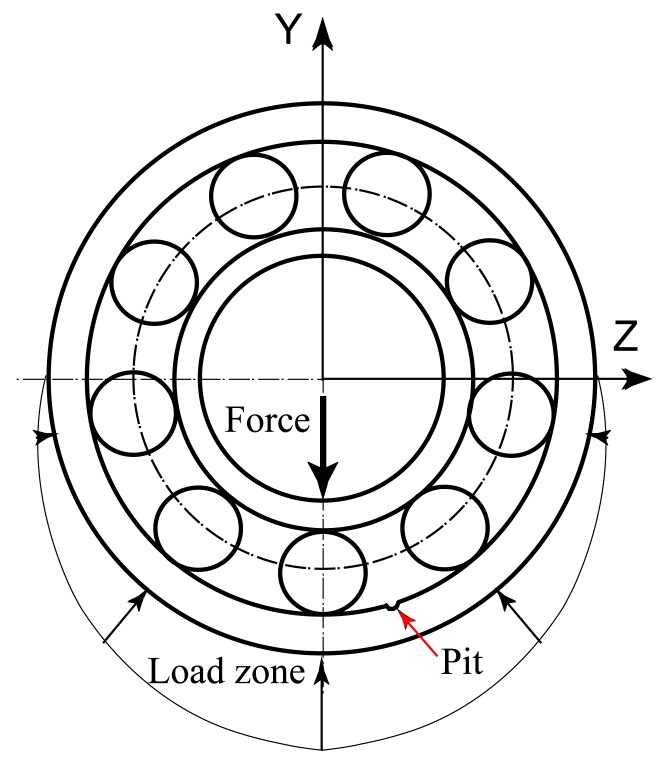
The localized defect on the outer ring.

The load is applied in the radial direction and there is a pit on the surface of the outer ring, as shown in Figure 12. When the rolling balls pass through the pit, the force impulses are generated. The size of the pit is assumed very small and just one rolling ball passes through the defective region at a certain time.

**Figure 13.**
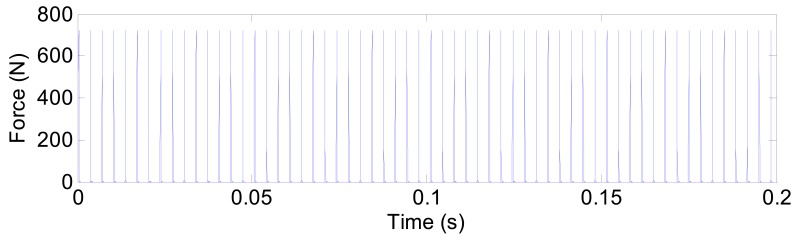
The successive force impulses.

The successive force impulses generated by the localized defect on the outer ring is shown in Figure 13. The time interval between two adjacent impulses is determined by the ball passing frequency for the outer raceway. In reality, there is a slight random fluctuation in the spacing between each force impulse because the load angle on each rolling element changes as the rolling elements enter and leave the load zone [25].

**Figure 14.**
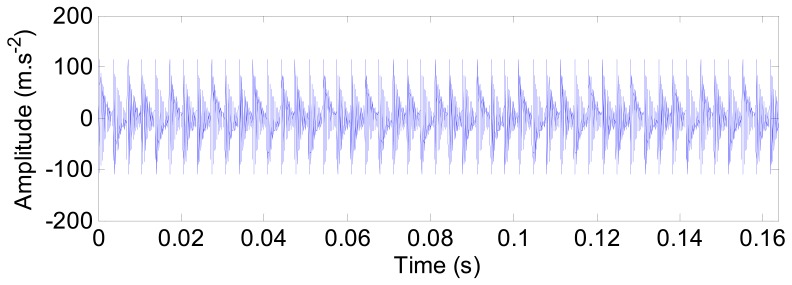
The impulse responses of the outer ring defect.

The impulse responses due to the outer ring defect are displayed in Figure 14. For each force impulse, a decay response is generated.

**Figure 15.**
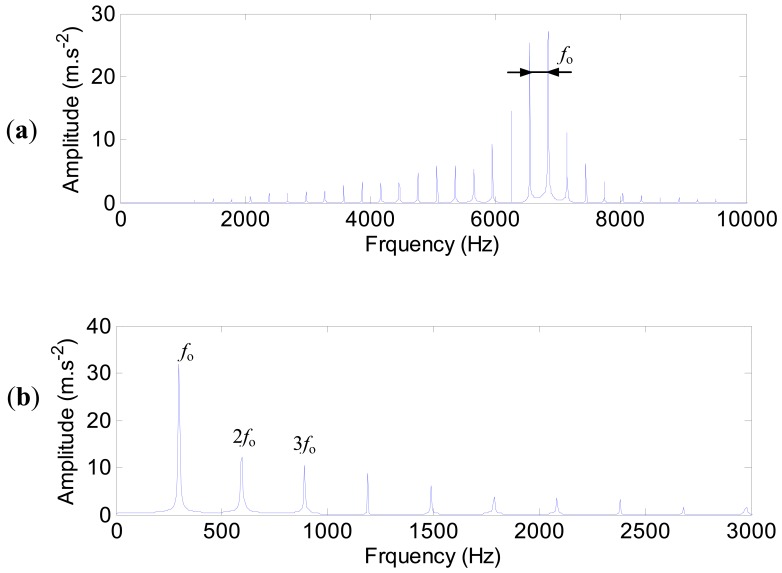
The characteristic frequencies of the outer ring defect. (**a**) The spectrum. (**b**) The envelop spectrum.

The characteristic frequencies of the outer ring defect are shown in Figure 15. The high harmonics of the defect frequency in the range of 4,000–8,000 Hz are quite clear (Figure 15(a)). The envelope spectrum (Figure 15(b)) is obtained by using the Hilbert transform technique. The defect frequency of the outer ring (298 Hz) and its harmonics are extracted clearly.

**Figure 16.**
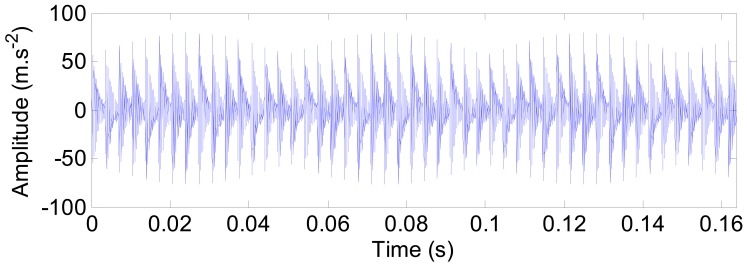
The amplitude modulated impulse responses of the outer ring defect.

For the spindle-bearing system, out-of-balance contributions at the shaft rotational frequency *f*_r_, is always evident as various sources (e.g., shaft mis-alignment) contribute to unbalanced rotation of the shaft. To include the out-of-balance effect, the vibration responses induced by force impulses are modulated in amplitude, as shown in Figure 16.

**Figure 17.**
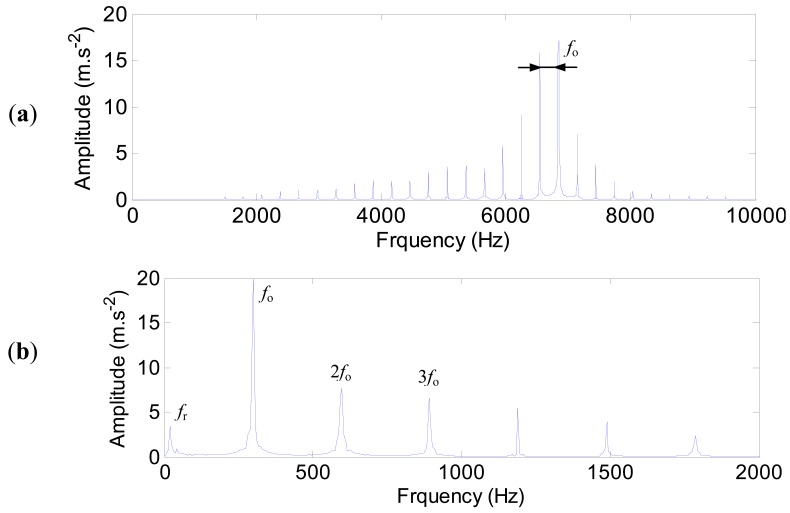
The characteristic frequencies of the outer ring defect with the out-of-balance effect. (**a**) The spectrum. (**b**) The envelop spectrum.

The characteristic frequencies of the outer ring defect with the out-of-balance effect are shown in Figure 17. Besides the defect frequency of the outer ring at 298 Hz and its harmonics, the rotating frequency *f*_r_ (20 Hz) is extracted as well in Figure 17(b).

**Figure 18.**
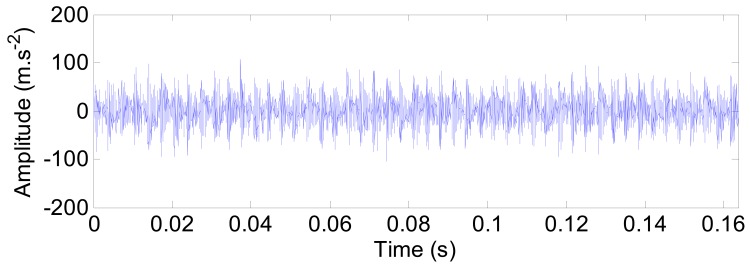
The amplitude modulated impulse responses of the outer ring defect with noise contamination.

For measurements in practice, the noise problem can never be avoided. For enhancing the simulation towards more realistic data, the impulse responses are contaminated with a normal distribution *N*(0,1) noise such that:

(16) y(t)=y(t)(1+w×N)

where *w* is the noise level. As an illustration, the amplitude modulated impulse responses of the outer ring defect with noise contamination is shown in Figure 18.

**Figure 19.**
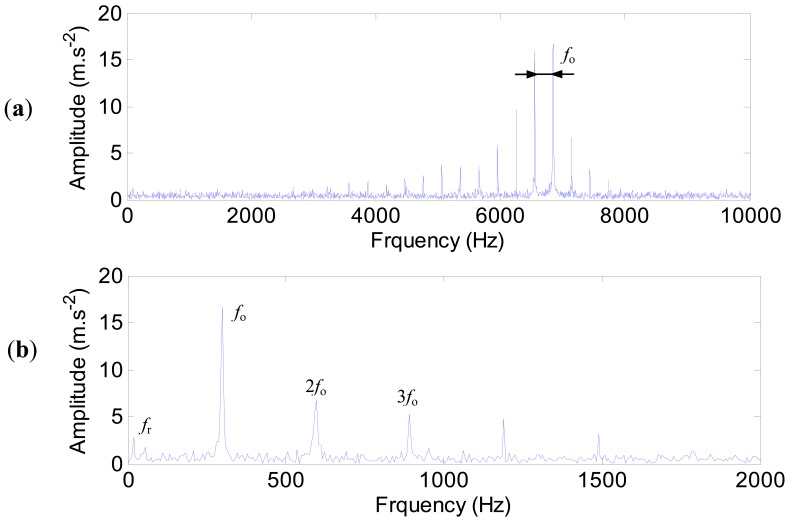
The characteristic frequencies of the outer ring defect with the out-of-balance and the noise contamination effects. (**a**) The spectrum. (**b**) The envelop spectrum.

The characteristic frequencies of the outer ring defect with the out-of-balance and the noise contamination effects are shown in Figure 19. Due to the noise, the spectra are not so clear as in Figure 17, and the amplitudes at the characteristic frequencies decrease. It can be predicted that when the signal-to-noise ratio is very low, the characteristic frequencies might be merged. Consequently, advanced signal processing techniques become indispensable to extract the bearing fault features from the raw signals contaminated by noise.

## 4. The Optimization of Sensor Placement

For the condition monitoring and diagnosis of the spindle bearings, vibrations produced due to defects are usually measured using the velocity transducer or accelerometers. However, the measured vibration responses are affected by the transmitting media between defects and sensors. To obtain a better monitoring effect, the optimization of the sensor position is necessary.

**Figure 20.**
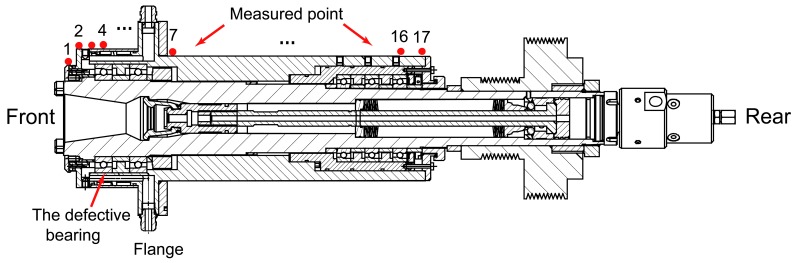
The sensor distribution on the test spindle.

In this study, the optimization scheme of the sensor position is carried out on the test spindle, as shown in Figure 20. The spindle is fixed on the machine tool at the flange by bolts. It is assumed that the defect occurs at the outer ring of the first bearing (HYKH61914). Since the spindle housing is stationary, seventeen measured points are chosen evenly on the surface of the spindle housing to detect the bearing fault. The measured point 7 is chosen on the fixed flange. The position of the measured point 4 is coincident with the defect bearing in axial direction. Next, the best measured point for detecting the bearing defect will be determined.

**Figure 21.**
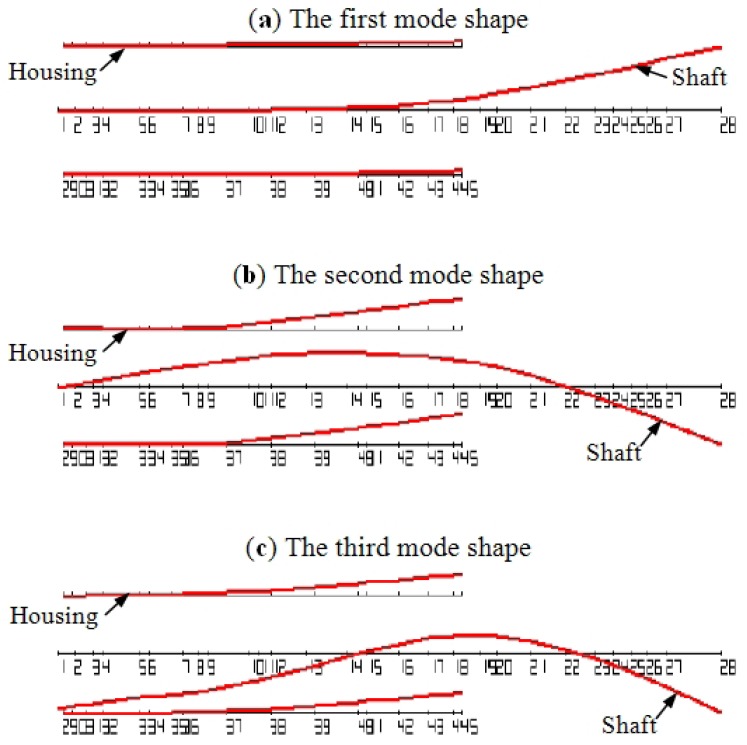
The first three mode shapes of the spindle (The flange is fixed).

It is well known that the displacement of a vibratory system due to the excitation is related with mode shapes. In Figure 21, the first three mode shapes of the test spindle are displayed. It can be seen that, the flexural mode of the shaft is the dominant vibration mode. For the housing, the displacement at the rear is much larger than that at the front. From the aspect of the mode shapes, the point 17 at the rear of the spindle housing may be the best choice to detect bearing defects.

On the other hand, it is better to monitor the vibration response signals at the nearest location to the defective bearing from experience [42]. From this point of view, point 4 may be the best position to mount the sensor, since it is the closest measured point to the defective bearing.

**Figure 22.**
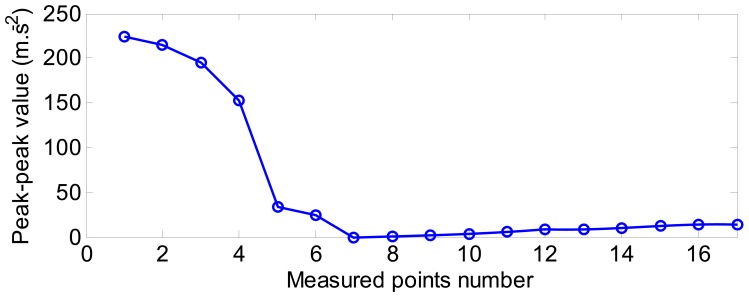
The peak-to-peak value of vibration responses.

The acceleration responses due to the localized defect are simulated by using the dynamic spindle-bearing model. The dynamic responses at different positions are simulated and then compared with each other to obtain an optimal sensor position. The peak-to-peak value of the vibration responses at each measured point is displayed in Figure 22. Since the measured point 7 is chosen on the fixed flange, the simulated vibration response is zero from the dynamic model. It is necessary to clarify that the vibration response at point 7 exists in reality and can be measured definitely. In our dynamic model, the boundary conditions are simplified and the flange is fixed rigidly on the ground. Therefore, the simulated vibration response at point 7 is zero. The simulation gives a different aspect: the amplitude of the vibration response at point 1 is the largest, which means that the point 1 is the best position to measure the vibration responses induced by the localized defect bearing. Neither point 4 nor point 17 will be chosen in practice.

Although the point 4 is the nearest point to the defective bearing, it is also close to the fixed flange which lowers the vibration response. For point 17, although the displacement of the mode shapes is the largest, the vibration energy decreases largely due to the long transfer path between the excitation and the response. It can be concluded that, the optimal sensor placement depends on the vibration modes under different boundary conditions, and the transfer path between the excitation and the response.

**Figure 23.**
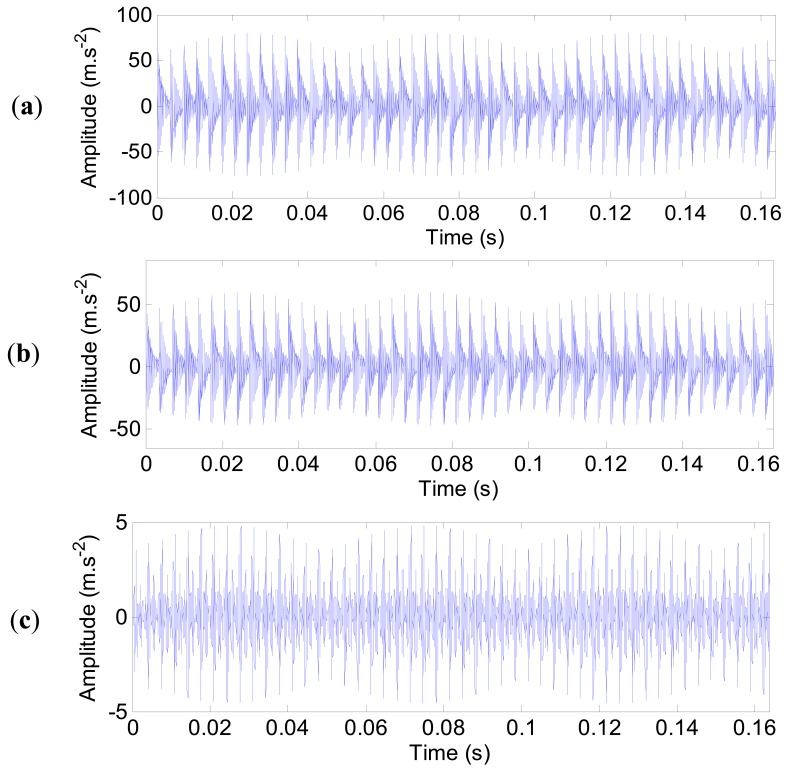
The noise-free impulse responses of the outer ring defect. (**a**) Point 1. (**b**) Point 4. (**c**) Point 17.

The impulse responses of the outer ring defect at three typical measured points (1, 4 and 17) are shown in Figure 23(a–c). The impulse responses are equally spaced. It is clear that the amplitude of the impulse responses is different. Here, the noise is not included.

**Figure 24.**
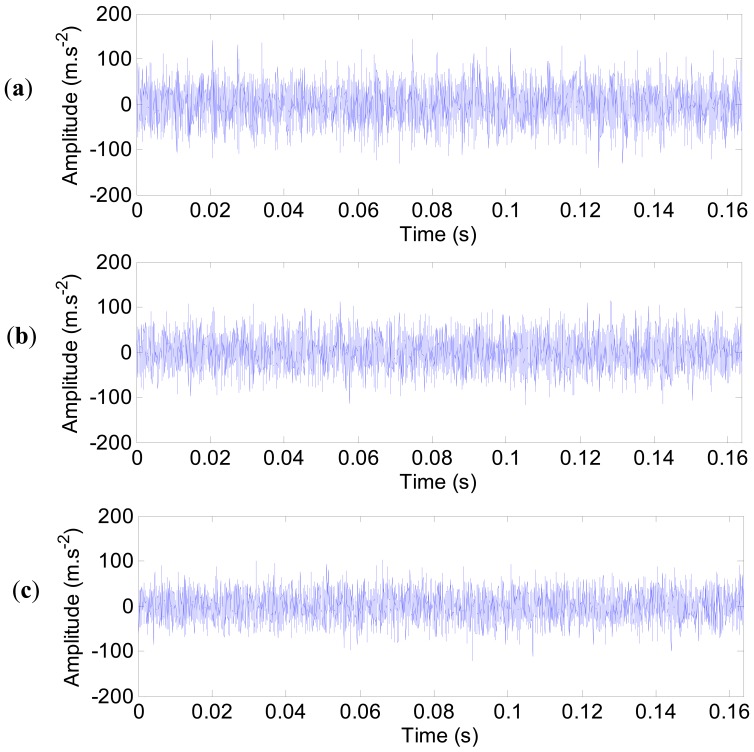
The impulse responses of the outer ring defect with noise. (**a**) Point 1. (**b**) Point 4. (**c**) Point 17.

Next, the noise contamination is included to enhance the simulation towards more realistic data. The impulse responses with noise contamination at the three measured points (1, 4 and 17) are displayed in Figure 24(a–c). It can be seen that the equally spaced impulse responses are submerged in the strong white noise. The signal-to-noise ratios (SNRs) of the impulse responses with noise are calculated. For the three measured points (1, 4 and 17), the SNRs are −1.52 dB, −4.61 dB and −25.87 dB, respectively.

**Figure 25.**
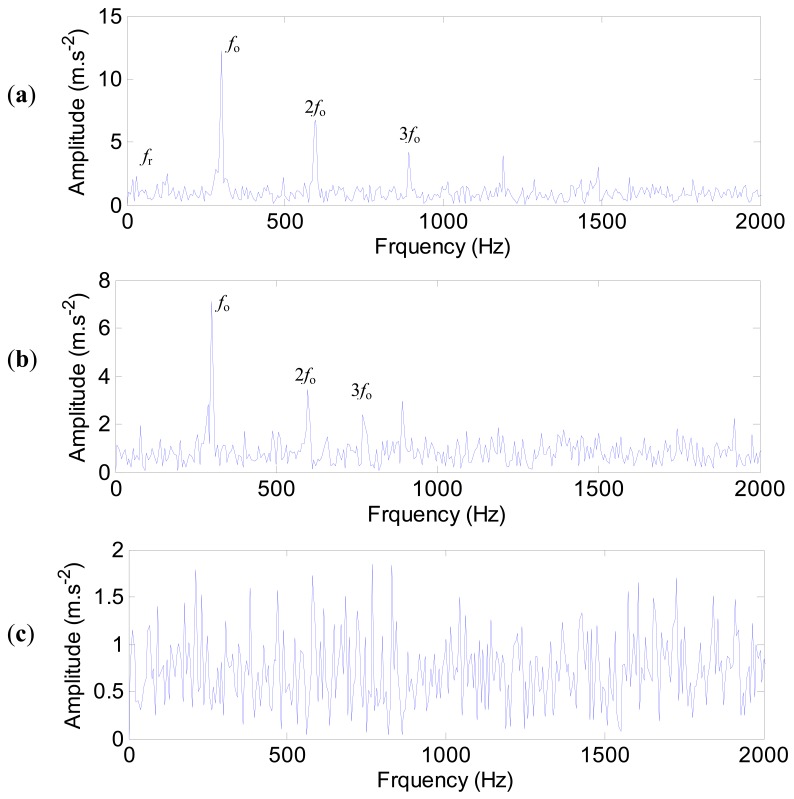
The envelop spectrum of the impulse responses with noise. (**a**) Point 1. (**b**) Point 4. (**c**) Point 17.

The envelope spectra of the impulse responses with noise are shown in Figure 25. For the measured point 1, the defect frequency of the outer ring (298 Hz) and its harmonics are extracted clearly. The rotating frequency *f*_r_ (20 Hz) is extracted as well (Figure 25(a)). For point 4 (Figure 25(b)), the defect frequency of the outer ring and its harmonics can still be extracted, but the information of the rotating frequency is lost. The worst case is the point 17 shown in Figure 25(c), where none characteristic frequency is extracted because of the low signal-to-noise ratio. Therefore, for the test spindle, if bearing defects occur at the first bearing, the point 1 is the best position to mount the sensor. This case study illustrates the significance of sensor placement optimization. If the sensor is mounted at improper position, the bearing faults may not be detected.

## 5. Conclusions

In the current study, the vibration response simulation and sensor placement optimization of machine tool spindles were investigated using an integrated FE model. The localized bearing defects in spindles were modeled visually, and vibration responses generated due to the outer ring defect were simulated as an illustration. The results have shown that:

1. The FE model of the spindle is capable of predicting the acceleration time responses due to the excitation. With the input excitation from an appropriate bearing fault model, the simulation of vibration response caused by bearing defects in machine tool spindles is feasible.
2. The noise decreases the amplitudes at the bearing characteristic frequencies in the envelop spectrum. If the signal-to-noise ratio is very low, the characteristic frequencies are submerged and cannot be detected from sensor signals.
3. The optimization of sensor placement in machine tool spindles depends on the vibration modes under different boundary conditions and the transfer path between the excitation and the response.

## Figures and Tables

**Table 1.** Parameters of the fault bearing.

**Type**	**Pitch diameter (mm)**	**Ball diameter (mm)**	**Ball numbers**	**Contact angle (**°**)**
HYKH61914	85	6.35	32	25
